# A Wide Passband Frequency-Selective Surface with a Sharp Roll-Off Band Using the Filtering Antenna-Filtering Antenna Method

**DOI:** 10.3390/ma17246131

**Published:** 2024-12-15

**Authors:** Yanfei Ren, Zhenghu Xi, Qinqin Liu, Jiayi Gong, Zhiwei Sun, Boyu Sima

**Affiliations:** 1 The 10th Research Institute of China Electronics Technology Group Corporation, No. 85 Yingkang West Road, Jinniu District, Chengdu 250102, China; swjturen@163.com; 2 Shanghai Radio Equipment Research Institute, Shanghai Academy of Spaceflight Technology, Shanghai 201109, China; xizhenghu@163.com; 3 Key Laboratory of Near-Range RF Sensing ICs and Microsystems (NJUST), Ministry of Education, School of Electronic and Optical Engineering, Nanjing University of Science and Technology, Nanjing 210094, China; irene98van@163.com (Q.L.); gongjiayi@njust.edu.cn (J.G.); 4 School of Microelectronics and Communication Engineering, Chongqing University, Chongqing 400044, China

**Keywords:** frequency-selective surface (FSS), filtering antenna (FA), high selectivity, sharp roll-off, reconfigurable

## Abstract

Frequency-selective surfaces (FSSs) have attracted great attention owing to their unique feature to manipulate transmission performance over the frequency domain. In this work, a filtering antenna-filtering antenna (FA-FA) FSS with a wide passband and double-side sharp roll-off characteristics is presented by inter-using the filtering antenna and receiving–transmitting metasurface methods. First, a dual-polarized filtering antenna element was designed by employing a parasitic band-stop structure with an L-probe feed. Then, the FA-FA-based FSS unit was constructed by placing two such filtering antennas back to back, with their feedings connected through metallic vias. Finally, the FSS with a wide passband and high selectivity was realized by arraying the FA-FA units periodically. The full-wave simulation results demonstrated that the designed FA-FA-based FSS had a wide passband from 13.06 GHz to 14.46 GHz with a flat in-band frequency response. The lower and upper roll-off bandwidths were sharp, reaching 1% and 1.2% of the center frequency. The proposed FA-FA-based FSS was fabricated and measured, achieving the coincident performance according to the theoretical prediction. The wideband band-pass FSS obtained a sharp double-side roll-off feature, which can be applied in various studies such as an antenna array, metasurface, communication, etc.

## 1. Introduction

A frequency-selective surface (FSS) is a special two-dimensional periodic structure which can produce selective responses of reflection, absorption or transmission to incident electromagnetic waves within a specific frequency range. As an important electromagnetic wave regulation technology, FSS is widely used in the microwave and millimeter-wave fields. With the development of 5G communication technology, the demand for high-selectivity FSS [1,2,3,4,5] has become significant. It can effectively suppress interference in signal transmission and improve the anti-interference ability of the system, thus playing a crucial role in modern wireless communication, radar systems and antenna design.

According to the differences in geometric structure, working mode and functionality, an FSS can be classified into several types. For example, it can be a single-layer FSS [6,7] and 3D FSS [8,9,10]; transmissive FSS and reflective FSS [11,12]; or conventional FSS, tunable FSS and phased-array FSS [13,14]. In recent years, the antenna–filter–antenna (AFA) structure has gradually become a new FSS design concept [15,16]. In previous FSS research, FSS was constructed through distribution parameter manipulation, which is accomplished by the design of the elements and the coupling between elements. This requires a complicated structural design. Owing to its ease of design and optimization, the antenna–filter–antenna (AFA) FSS method [17,18,19], which integrates the antenna, filter, and antenna modules, has recently attracted attention. However, AFA-FSSs have some drawbacks such as (i) a comparatively higher profile since a filter is introduced, (ii) a mutual coupling between the filter and the antenna that may impair FSS performance, and (iii) the FSS is difficult to be actively controlled because the filter is positioned inside the antennas, making it hard to introduce the active devices. Furthermore, in high-frequency or wideband applications, the system is still limited by the size and frequency response constraints. In this study, the receiving–transmitting metasurface and filtering antenna approaches were applied to achieve the FSS design. Because the filter module was built within the antennas, the FSS profile was reduced; meanwhile, it was simple to introduce the active devices for modulating transmission features.

The filtering antenna [20,21,22,23,24] is an innovative design that combines the filter function with antenna performance, and can achieve higher selectivity and flexibility during signal transmission. By optimizing the filter design, the filtering antenna can provide excellent gain within a specific frequency range, while effectively suppressing unnecessary frequency components. Compared with the filtering antenna, the AFA has a larger physical size and weight, which is not conducive to miniaturized devices and makes integration difficult. The filtering antenna not only improves the signal clarity, but also expands the application range of the system. In particular, in a complex electromagnetic environment, it shows significant advantages. The paper presents an FSS design method based on the FA-FA structures to realize wide-passband and double-side sharp roll-off characteristics [25,26]. Firstly, a dual-polarized filtering antenna element based on the parasitic band-stop structure with an L-probe feed was designed. Then, two of these filtering antennas sharing a common floor were placed back to back and the feed metal posts were connected through holes in the floor to form the basic FA-FA unit. Finally, a dual-polarized FSS with wide passband and high selectivity was realized by arraying the above FA-FA unit. This design can effectively improve the frequency selectivity while maintaining a low profile and polarization stability. In addition, this structure was convenient for integrating active devices, thereby achieving reconfigurable functions [27,28] and meeting the requirements of different application scenarios. This flexibility allows our design to have broad application prospects in future communication systems.

This research inter-applies two focused research issues: the filtering antenna and receiving–transmitting metasurface, resulting into a feasible and effective strategy for the wideband and sharp roll-off FSS design. Compared with classical surface-type FSS, FA-FA-based FSS avoids complicated design and optimization of the structure dimensions; compared with AFA FSS, FA-FA-based FSS integrates the filter device within antennas, which can reduce the profile and make it easier to introduce the active devices. The designed FSS also has some advantages in performance, such as a wider bandwidth, lower profile configuration and narrower double-side roll-off bandwidth. Furthermore, it also provides an opportunity for the reconfigurable control of the transmission features since our FSS can easy load the active devices. This paper is organized as follows: Section 2 presents the design principles of the proposed FSS. The elements of the FSS are detailed, systematically analyzed and simulated. Section 3 present the measurement setup and experimental results of the band-pass FSS. Last, Section 4 concludes the paper.

## 2. Design and Analysis of FSS

### 2.1. Design Principle

**Figure 1:**
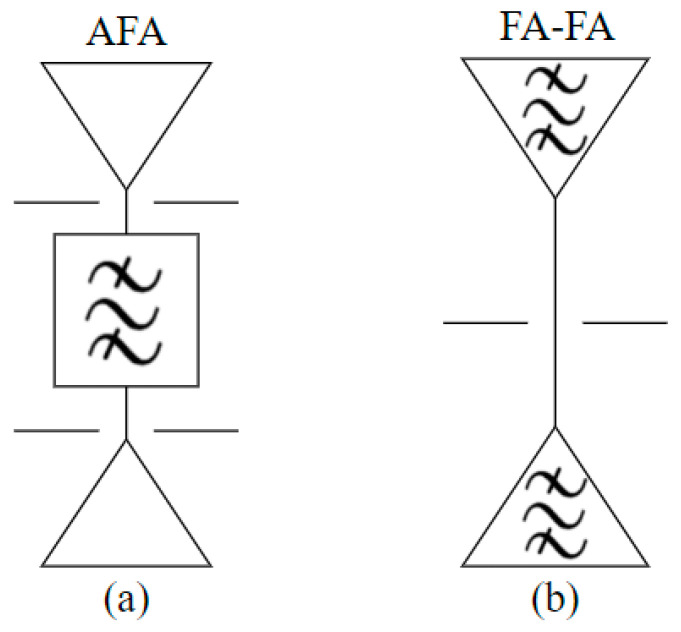
Schematic design of (**a**) the AFA-based FSS and (**b**) the FA-FA based FSS.

As shown in Figure 1, compared with some AFA-based FSS units in which the antenna and filter functions are designed separately and later cascaded by matching circuits, in this paper, the antenna and filter functions were integrated in a single passive device, which realizes both the radiation and filter functions simultaneously, and on this basis, the FA-FA-based FSS was designed.

**Figure 2:**
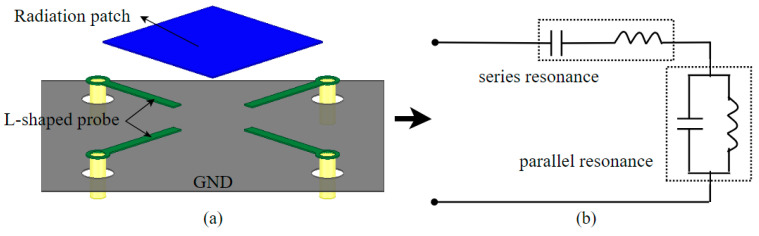
L-shaped probe feed patch antenna: (**a**) schematic structure and (**b**) equivalent circuit diagram.

The basic structure of the filter antenna was chosen to be a patch antenna fed by an L-shaped probe, and the schematic structure is shown in Figure 2a. Its equivalent circuit diagram is shown in Figure 2b, and vertical and horizontal components can be equivalent to the integrated circuit of series resonance and parallel resonance. The feeding method is an important aspect that affects the performance of the microstrip antenna, and the common feeding methods mainly include slit-coupled feeding, probe feeding and microstrip line feeding. Among them, the probe feed is one of the more widely used feed methods. However, for the patch antenna with a thick dielectric layer, when the probe is longer, it will lead to the inductive enhancement of the antenna input impedance and affect the bandwidth of the antenna. In this design, the L-shaped probe feed can effectively overcome this effect.

The radiation mechanism of the L-probe feed can be explained as the vertical part of the probe produces inductive impedance with the patch, and the horizontal part produces capacitive impedance with the patch, which interacts to produce resonance, making the antenna exhibit wide-passband characteristics. An alternating electric field exists on the L-probe, which results in a varying magnetic field, and the direction of the field is perpendicular to the direction of the electric field, which can be expressed in terms of the pointing of the horizontal arm of the probe. At the same time, the magnetic lines of force passing perpendicularly through the patch will also produce a variable electric field. It is radiated after reflection by the metal floor.

**Figure 3:**
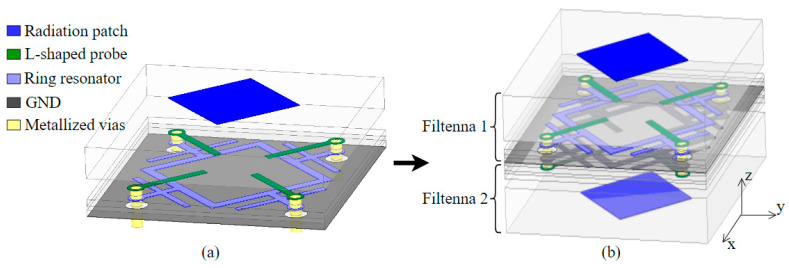
Three-dimensional view of (**a**) the filtering antenna and (**b**) FA-FA-based FSS unit.

The brief structure of the FA-FA-based FSS element is shown in Figure 3. The filtering antenna is shown in Figure 3a, which is made of a patch antenna (in blue), L-type feeding probe (in green), ring resonator (in purple) and background (in gray), from top to bottom. The feeding was positioned between the patch and ring resonator to receive or transmit the EM waves. Figure 3b shows the FA-FA-based FSS element which was constructed by two filtering antennas. The two antennas were positioned back to back, connected by the vias at the terminals of the feeding of the antennas. The antennas shared the background. When electromagnetic waves were incident to one side of the FSS, the filter antenna array on that side received the energy and transmitted it to the filter antenna array on the other side to complete the outgoing emission.

### 2.2. The FA-FA-Based FSS

**Figure 4:**
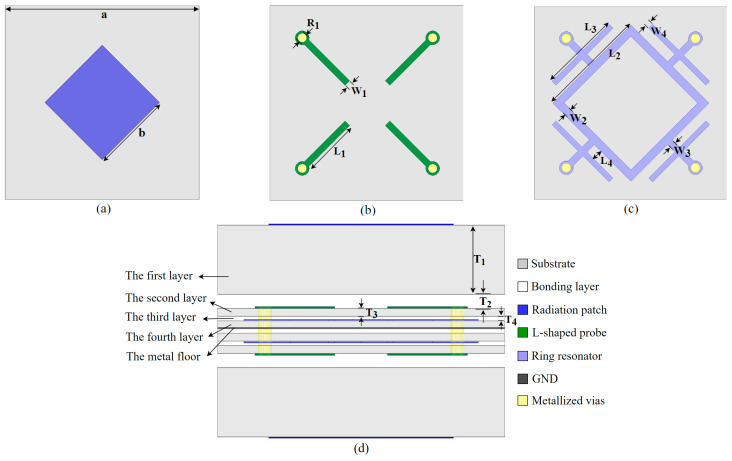
Unit structure of FA-FA-based FSS: (**a**) top view of first layer, (**b**) second layer, (**c**) fourth layer, and (**d**) side view.

The FSS proposed in this paper employs a multilayer structure, and since the structure is highly symmetric, only the composition of the upper half is presented in detail. As shown in Figure 4d, the first dielectric layer is made of F4B sheet with a dielectric constant ε_r_ = 2.65 and thickness of 1.5 mm. The second and fourth layer ere also made of F4B sheet with a dielectric constant ε_r_ = 2.65, but with a thickness of 0.17 mm. The third layer was the adhesive layer, and an FR4 sheet with a thickness of 0.1 mm was used. The length and width of all dielectric plates were 4.9 mm. The radiation patch was printed on the upper surface of the first dielectric plate, as shown in Figure 4a. The bent L-shaped metal arm was printed on the upper surface of the second layer of dielectric plate, as shown in Figure 4b, which can couple with the radiation patch and gather energy at resonance. The metal structure printed on the upper surface of the fourth dielectric plate is shown in Figure 4c. The square ring was equivalent to paralleling the parasitic structure underneath the L-shaped metal arm so that it can couple with the metal post to introduce both inductance and capacitance with each metal layer to generate a strong resonance. By controlling the length and width of the metal square ring, the FSS down-roll bandwidth can be controlled. Furthermore, the metal strips connected in parallel at the connection between the square ring and the metal post can optimize the high-frequency out-of-band rejection of the FSS.

In order to verify the performance parameters of the proposed FSS, its cell structure was simulated and optimized with the help of Ansys-HFSS 2023R1 software, and the optimized structural dimensional parameters are shown in Table 1.

**Figure 5:**
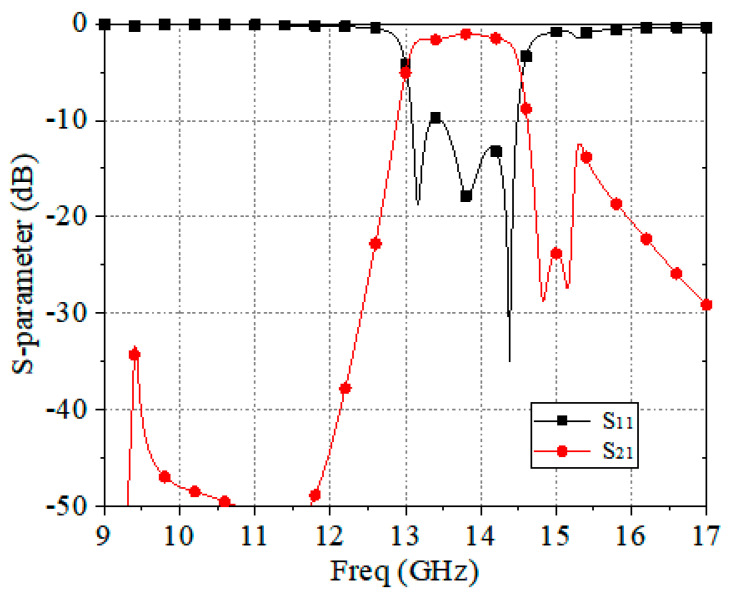
Simulated magnitude of the transmission of the proposed FA-FA-based FSS unit under normal incidence.

Due to the high symmetry of the proposed FSS, the designed FSS had good polarization stability at the incidence of electromagnetic waves with different polarizations, and there existed a good consistency in the S-parameter frequency response. The transmission and reflection coefficients at the vertical incidence of TE waves are shown in Figure 5. The proposed FSS had a good polarization stability at the incidence of electromagnetic waves with different polarizations, and there existed a good consistency in the S-parameter frequency response. The simulation results show that the proposed FSS had a passband from 13.06 GHz to 14.46 GHz and a flat in-band frequency response. The lower roll-off bandwidth and upper roll-off bandwidth reached 1% and 1.2% of the center frequency, respectively, to achieve a sharp roll-off skirt. The out-of-band rejection was also good.

**Figure 6:**
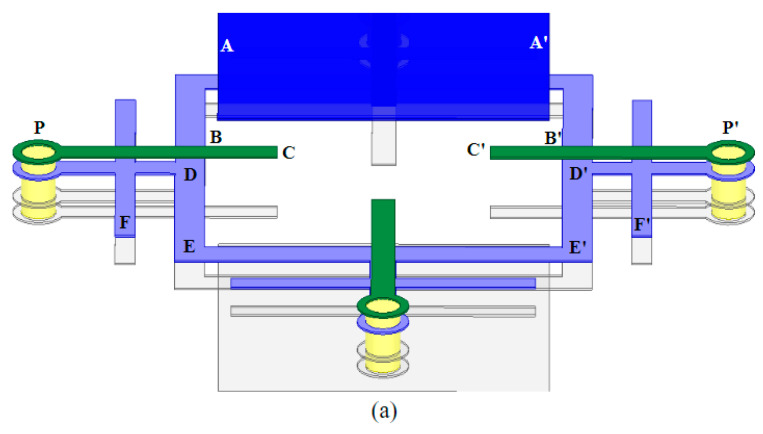

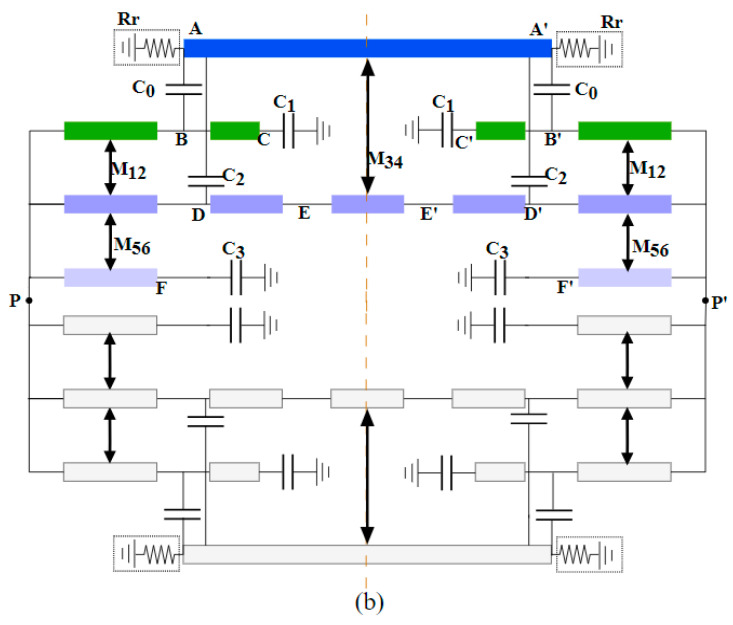
(**a**) Partial structure of the FSS and (**b**) the corresponding simplified equivalent transmission line model.

The filtering characteristics of the FSS were further investigated, and a simplified equivalent transmission line model was constructed to analyze the structure of each layer of the designed FSS unit, as shown in Figure 6. The FSS unit was designed to be a microstrip line structure, and the coupling effect can be equivalent to the capacitive effect. And according to the microstrip line theory, the coupling effect in the microstrip line structure can be equated to the capacitance effect. When an electromagnetic wave is incident on the FSS cell, the energy radiated into free space is equivalently represented by two radiation resistances Rr. The second layer of the curved L-shaped metal arm is represented by capacitance C_0_ between it and the top layer of patch A. The fourth layer between the metal structure and the top layer patch A is represented by the capacitance C_2_. The coupling effect between the bent L-shaped metal arm (P-B) and the square ring (P-D) is represented by the mutual coupling coefficient M_12_. The coupling effect between the square ring (E-E’) and the edge of the patch (A-A’) is represented by the mutual coupling coefficient M_34_. The coupling effect between the metal strip (F) and the square ring (P-D) is represented by the mutual coupling coefficient M_56_. The validation results show that good out-of-band rejection capability of the designed FSS can be realized by adjusting these parameters.

**Figure 7:**
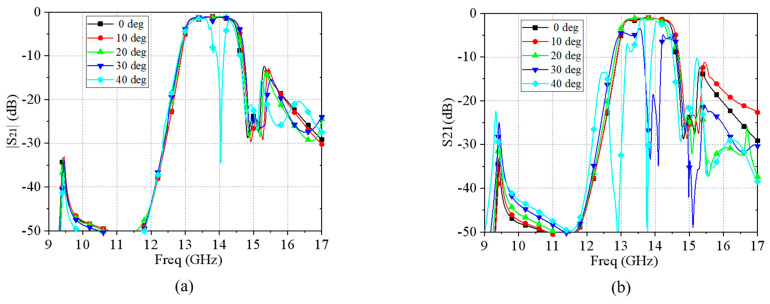
S-parameters for different incidence angles under (**a**) TE polarization and (**b**) TM polarization.

Based on the analysis of angular stability, the simulated S-parameters for two different polarizations of electromagnetic waves obliquely incident to the FSS are given in Figure 7a,b, respectively. Overall, the stability under TE polarization is good and can maintain good angular stability over the 0°–30° incidence angle range.

### 2.3. Design and Simulation of a Reconfigurable ACA FSS

**Figure 8:**
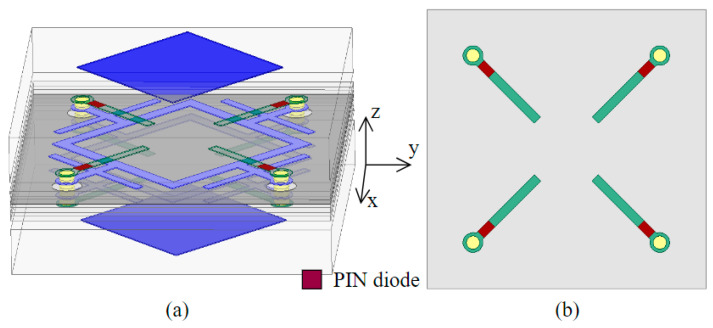
(**a**) The 3D diagram of the reconfigurable AFA FSS unit structure and (**b**) the top view of the second layer.

As discussed above, one of the advantages of our proposed FA-FA-based FSS is the convenience for the realization of reconfigurable manipulation functions by adding active components in the circuit lines. To give a preliminary verification, a reconfigurable design based on the proposed ACA FSS was also implemented. The schematic of the proposed reconfigurable unit cell is shown in Figure 8.

**Figure 9:**
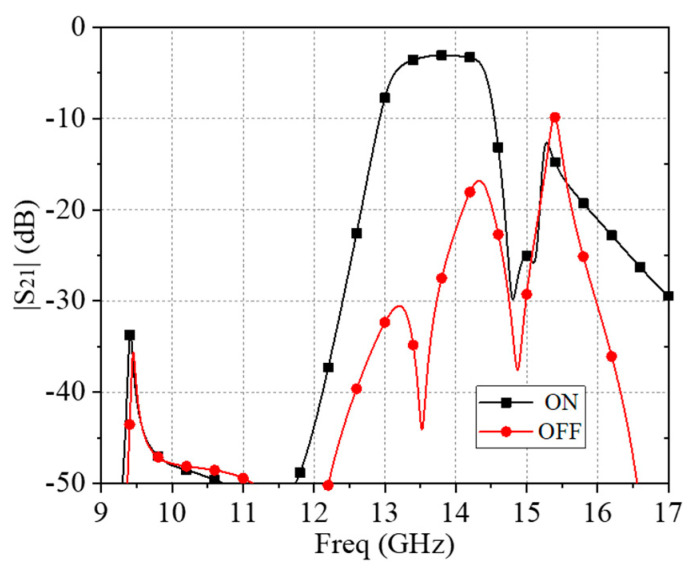
Simulated magnitude of the transmission of the proposed reconfigurable AFA FSS unit under normal incidence.

Four PIN diodes were inserted in the terminal of each branch of the feeding lines on the second dielectric layer of the original structure. The air between the first and second layer provided the necessary space for loading PIN diodes. The diode was approximately modeled as a 2.1 Ω resistor for the ON-state and a parallel circuit of a 0.17 pF capacitor and a 3 kΩ resistor for OFF-state according to Ref. [21]. To verify the performances of the proposed reconfigurable ACA FSS unit cell, the full-wave simulation was implemented by the HFSS 2023R1 software. The comparison of the simulated transmission magnitude of designed unit cell with PIN diodes in ON state and OFF state is given in Figure 9. As seen, the reconfigurable ACA FSS kept bandpass characteristics when the diodes were in the ON state. The −3 dB bandwidth of the passband was from 13.07 GHz to 14.46 GHz with an insertion loss of 3.1 dB, while it performed total reflection when the diodes were in the OFF state. Notably, this was only a preliminary verification, so bias lines were not added in the simulation at present. For a more realistic situation, bias lines should be considered and suitably designed to minimize the impact on the EM waves as much as possible. As shown in Figure 9, the transmission decreased to around −3 dB in the ON case, which was caused by the loss of employed PIN diodes. The diodes used here were BAR64, which have a 2.1 Ω resistance at this working band. This deterioration can be solved by replacing the type of diode or optimizing the introduced position.

## 3. Fabrication and Measurement

**Figure 10:**
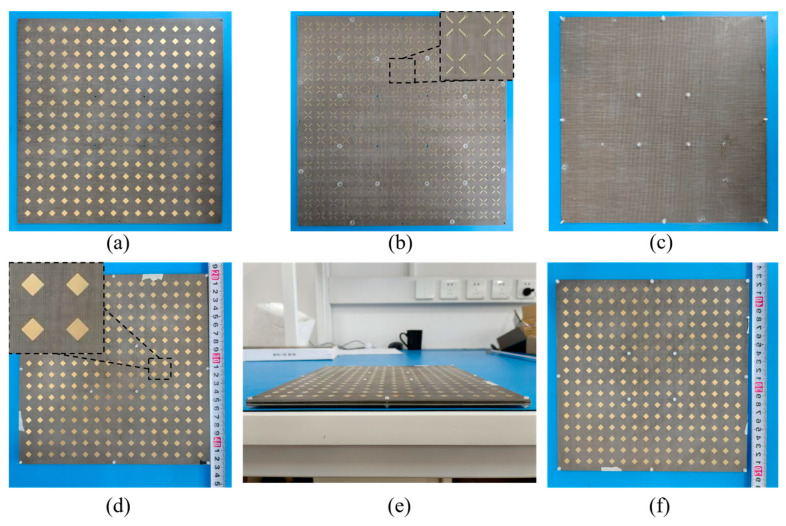
The fabrication photograph of the proposed FA-FA-based FSS prototype. (**a**) Front view of the upper plate. (**b**) Front and partial enlarged view of the middle plate. (**c**) Front view of the lower plate. (**d**) Front and partial enlarged view of the assembled prototype. (**e**) Side view of the assembled prototype. (**f**) Back view of the assembled prototype.

We designed and fabricated the prototype of the proposed frequency-selective surface (FSS). As shown in Figure 10, the FSS consisted of three layers: upper, middle and lower, which were constructed from three individual dielectric plates. To accommodate the metallized vias required in the middle layer, we utilized multiple nylon post screws combined with gaskets for compression. The dielectric plates were made of F4B, with a dielectric constant of 2.65. The single-layer dielectric plate was formed by bonding and stacking multiple layers of F4B, with a thickness of 0.17 mm. The adhesive layer between the F4B layers was made of FR4 with a thickness of 0.1 mm. A metal pattern was fabricated on the F4B plate with a metal layer thickness of 0.035 mm. The overall dimensions of the FSS prototype were 227.6 mm × 227.6 mm × 4.62 mm and consisted of 16 × 16 units.

**Figure 11:**
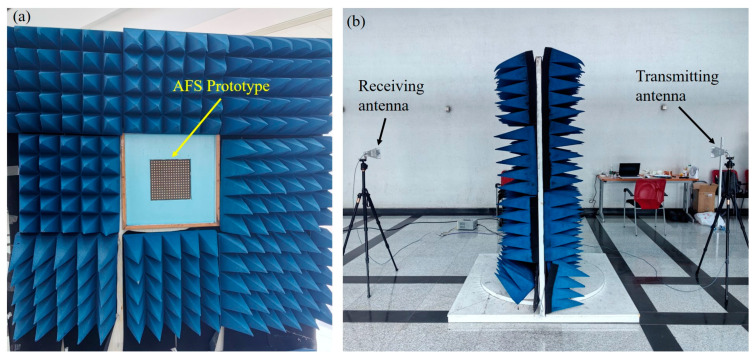
Measurement setup: (**a**) prototype in the measurement window and (**b**) the whole test environment.

The far-field transmission of the FSS was measured in an open space. In the experiment, two standard horn antennas were placed on either side of the FSS, serving as transmitting and receiving antennas, as shown in Figure 11. The test FSS prototype was positioned at the center of the measurement window, perpendicular to the ground, surrounded by absorbing materials to minimize scattering interference and surface reflections. Two standard horn antennas, operating in the frequency range of 2–18 GHz were mounted on tripods horizontally opposite to each other and perpendicular to the FSS prototype. The horn antennas were connected to two ports of a vector network analyzer (Agilent N9918A, which is manufactured by Keysight Technologies (formerly Agilent) in Santa Rosa, CA, USA), ensuring that the distance between the horn antennas and the sample satisfied the far-field conditions of the NRL standard. By rotating the platform equipped with the FSS prototype and changing the polarization of the horn antennas, the FSS transmission responses for TE and TM waves were measured, respectively, at incident angles of 0° and 30°.

**Figure 12:**
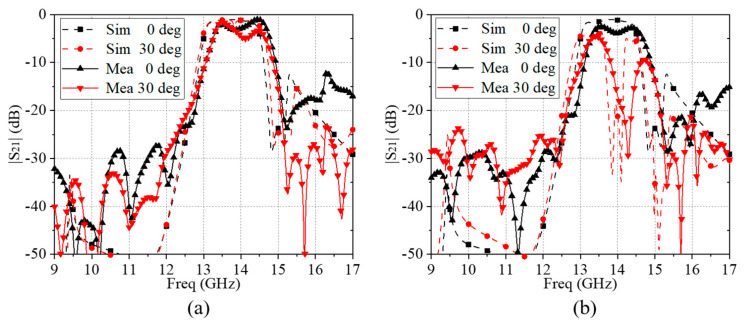
Comparison of the simulated and measured transmission results of the proposed FSS: (**a**) TE and (**b**) TM.

As illustrated in Figure 12, the measured transmission results of the FSS prototype under different incident angles and polarizations were compared with the simulated ones. The measured results for both the TE and TM polarizations demonstrated good agreement with the simulated ones, with rapid declines in the double-sided bandwidth. The measured results for the TE and TM waves at normal incidence were consistent with the simulated results, except minor deviations in frequency and amplitude. Similarly, the measured results for the TE wave at a 30° incidence aligned closely with the simulated ones. For the TM wave at a 30° incidence, the overall trend remained comparable to the simulated data, while the amplitude of the second peak showed a noticeable reduction. These findings indicate minimal discrepancies between the experimental results and simulated ones across the operating frequency band. The slight variations and losses observed are attributed to the actual experimental conditions, imperfect experimental design and potential errors arising from the manufacturing and measurement processes of the physical samples.

In summary, our findings indicate that the actual measurement results and the full-wave simulation results exhibited similar overall trends and effects. Despite some possible experimental discrepancies in the results, the proposed frequency-selective surface (FSS) demonstrated several advantages, including a flat transmission amplitude within the passband, steep roll-off on both sides, favorable rectangular characteristics and ease of integration with active components.

Compared to the research in [2,4,5,19], the proposed FA-FA structure obtained a wider bandwidth around 62%, at a relatively high center frequency of 14 GHz, as shown in Table 2. Meanwhile, the FSS profile was significantly reduced to 0.21λ_0_, which equals to 0.66λ_0_ and 0.33λ_0_ in [8,9], respectively. This low-profile design is particularly crucial for lightweight and compact devices. In addition, compared with the classical surface-type FSS, the FA-FA-based FSS avoids a complicated design and parameter optimization of the element dimensions. It also achieves a feasible and simple method for modulating the FSS transmission features, which can improve active and reconfigurable performance.

## 4. Conclusions

We present a frequency-selective surface (FSS) design method based on the filtering antenna-filtering antenna (FA-FA) structure to realize wide-passband and double-side sharp roll-off characteristics. The unit of the proposed FA-FA structure was composed of two dual-polarized filtering antennas connected back to back. A wide passband and high selectivity were realized by arraying the above FA-FA unit. The full-wave simulation results demonstrated that the proposed FSS has a passband from 13.06 GHz to 14.46 GHz with flat in-band frequency response. The lower and upper roll-off bandwidth reached 1% and 1.2% of the center frequency. A reconfigurable FA-FA-based FSS was also designed by loading PIN diodes, and were proven by the simulation results. The prototype of the proposed passive FA-FA-based FSS was fabricated and good performance was obtained. Both the simulation and measurement demonstrated that the proposed FA-FA-based FSS had wide-passband and fast double-side roll-off characteristics. Due to its good performance in bandwidth, high selectivity and low profile, our designed FA-FA-based FSS has good application prospects in radome, electromagnetic shielding, communication anti-interference, etc.

## Figures and Tables

**Table 1:** Optimized structural parameters of the FSS unit.

**Parameter**	a	b	L_1_	L_2_	L_3_	L_4_	W_1_	W_2_
**Value/mm**	13.6	4.9	3.6	6.3	4.59	1.8	0.3	0.45
**Parameter**	W_3_	W_4_	R_1_	T_1_	T_2_	T_3_	T_4_	
**Value/mm**	0.39	0.3	0.26	1.5	0.3	0.17	0.1	

**Table 2:** Performance comparison with some reported works.

Ref.	*f*_0_ (GHz)	−3 dB FBW	Profile Height (λ_0_)	Lower Roll-Off Bandwidth	Upper Roll-Off Bandwidth	Pol.
[1]	2	155.7%	0.029	1.8% *f*_0_	7.3% *f*_0_	Dual
[2]	10	35%	0.31	3.89% *f*_0_	1.2% *f*_0_	Dual
[4]	25.6	20.2%	0.276	4.25% *f*_0_	3.16% *f*_0_	Single
[5]	3.57	29%	0.05	9.8% *f*_0_	8.7% *f*_0_	Dual
[8]	12	98.5%	0.66	6.51% *f*_0_	1.3% *f*_0_	Dual
[9]	9.63	78.5%	0.33	6.4% *f*_0_	4.7% *f*_0_	Dual
[19]	10	11.5%	0.17	2.6% *f*_0_	6.0% *f*_0_	Dual
This work	14	62%	0.21	1% *f*_0_	1.2% *f*_0_	Dual

Notice: *f*_0_ is the center frequency and λ_0_ is the free-space wavelength corresponding to the center frequency.

## Data Availability

The data that support the findings of this study are available from the corresponding author (Z.S.) upon reasonable request due to privacy.
